# Nuclear membrane ruptures, cell death, and tissue damage in the setting of nuclear lamin deficiencies

**DOI:** 10.1080/19491034.2020.1815410

**Published:** 2020-09-10

**Authors:** Natalie Y. Chen, Paul H. Kim, Loren G. Fong, Stephen G. Young

**Affiliations:** a Department of Medicine, University of California, Los Angeles, CA, USA; b Department of Human Genetics, University of California, Los Angeles, CA, USA; c Department of Molecular Biology Institute, University of California, Los Angeles, CA, USA

**Keywords:** Nuclear membrane rupture, nuclear envelope, nuclear lamina, nuclear lamins, B-type lamins

## Abstract

The nuclear membranes function as a barrier to separate the cell nucleus from the cytoplasm, but this barrier can be compromised by nuclear membrane ruptures, leading to intermixing of nuclear and cytoplasmic contents. Spontaneous nuclear membrane ruptures (*i.e*., ruptures occurring in the absence of mechanical stress) have been observed in cultured cells, but they are more frequent in the setting of defects or deficiencies in nuclear lamins and when cells are subjected to mechanical stress. Nuclear membrane ruptures in cultured cells have been linked to DNA damage, but the relevance of ruptures to developmental or physiologic processes *in vivo* has received little attention. Recently, we addressed that issue by examining neuronal migration in the cerebral cortex, a developmental process that subjects the cell nucleus to mechanical stress. In the setting of lamin B1 deficiency, we observed frequent nuclear membrane ruptures in migrating neurons in the developing cerebral cortex and showed that those ruptures are likely the cause of observed DNA damage, neuronal cell death, and profound neuropathology. In this review, we discuss the physiologic relevance of nuclear membrane ruptures, with a focus on migrating neurons in cell culture and in the cerebral cortex of genetically modified mice.

## Early descriptions of nuclear membrane ruptures

Nuclear membrane ruptures were observed in 2001 by de Noronha *et al*. [1] while working to elucidate how Vpr, a multifunctional HIV-1 protein, affects nuclear–cytoplasmic trafficking. They found that overexpression of Vpr in HeLa cells resulted in herniations of nuclear chromatin (nuclear blebs) and noted that blebs appeared in segments of the nuclear envelope that were deficient in lamin C and nuclear pore complexes. Vpr overexpression was accompanied by ruptures of nuclear membranes, resulting in the escape of nuclear contents into the cytoplasm. They assumed that nuclear membrane ruptures originated from the nuclear blebs. In 2006, Cohen *et al*. [2] examined parvovirus-infected fibroblasts and observed evidence of nuclear membrane ruptures. Immunofluorescence microscopy studies with antibodies against lamins A/C revealed gaps in the nuclear lamina, and electron microscopy studies identified discontinuities in the nuclear envelope [2].

Subsequently, nuclear membrane ruptures were identified in human fibroblasts harboring missense or nonsense mutations in *LMNA* (the gene for lamin A and lamin C) and in cancer cell lines (osteosarcoma cells, HeLa cells) [3–5]. In 2011, De Vos *et al*. [3] observed spontaneous nuclear membrane ruptures in fibroblasts from patients with *LMNA* missense mutations (including mutations causing familial partial lipodystrophy, cardiomyopathy, and a progeroid disorder). In 2013, Tamiello *et al*. [4] observed nuclear membrane ruptures in fibroblasts from a two-year-old child with a progeroid disorder resulting from compound heterozygosity for p.T528M and p.M540T mutations in *LMNA*. The fibroblasts in the latter study contained a nuclear-localized yellow fluorescent protein (EYFP-NLS), and nuclear membrane ruptures were visualized by the escape of the EYFP-NLS into the cytoplasm. Vargas *et al*. [5] observed spontaneous nuclear membrane ruptures in cultured osteosarcoma and HeLa cells, which have baseline abnormalities in nuclear shape and where nuclear lamin proteins can be distributed unevenly along the nuclear rim. Using osteosarcoma cells that had been transfected with GFP containing a nuclear localization signal (GFP_3_-NLS), they observed, by videomicroscopy, nuclear membrane ruptures in ~8% of the cells, with most ruptures undergoing repair within 30 min (allowing for return of GFP_3_-NLS into the nucleus). Most cells with a nuclear membrane rupture had a nuclear bleb. The frequency of nuclear membrane ruptures increased to ~25% when the cells were transiently transfected with an siRNA cocktail against lamin A/C, lamin B1, and lamin B2  or when the cells were stably transfected with a short hairpin RNA (shRNA) against lamin B1. Transfection of the latter cells with a lamin B2 expression vector reduced the frequency of nuclear membrane ruptures [5].

## Nuclear membrane rupture frequency increases when cells are subjected to mechanical stress

Several studies have examined the impact of mechanical stress on nuclear membrane ruptures [6–8]. Harada *et al*. [9] found that serum-driven migration of lung carcinoma–derived A549 cells through 3-μm pores resulted in a large increase in caspase 3–positive cell death in the setting of lamin-A knockdown. In 2016, Raab *et al*. [6] found, by videomicroscopy, that bone marrow–derived mouse dendritic cells expressing a nuclear-localized GFP (NLS-GFP) exhibited nuclear membrane ruptures as they migrated through collagen-filled channels containing 5-μm constrictions and when the cells migrated within a mouse ear explant. During cell migration, the nuclei became deformed, followed by nuclear membrane ruptures, evident from reduced NLS-GFP in the nucleoplasm and escape of NLS-GFP into the cytoplasm. They also observed nuclear membrane ruptures in human monocyte-derived dendritic cells, HeLa cells, and retinal pigment epithelial (RPE-1) cells. Nuclear protrusions often preceded the nuclear membrane rupture. To define the site of nuclear membrane rupture, Raab *et al*. [6] examined the localization of a GFP-tagged cytoplasmic DNA-binding probe (cyclic guanosine monophosphate-adenosine monophosphate synthase; cGAS) in RPE1 cells as the cells migrated across narrow constrictions. They identified GFP-cGAS foci at the leading edge of the cell – the same site where nuclear protrusions were observed. They also found that nuclear membrane ruptures were associated with double-stranded DNA breaks [6]. When RPE1 cells with a lamin A/C knockdown were forced to traverse narrow constrictions, nuclear membrane ruptures were accompanied by reduced cell survival.

At the same time, Denais *et al*. [7] observed nuclear membrane ruptures in human fibroblasts, breast cancer cells, and fibrosarcoma cells migrating in a microfluidic device with 2-μm constrictions. They documented nuclear membrane ruptures by the escape of NLS-GFP into the cytoplasm and by visualizing the distribution of a GFP containing a nuclear export signal (NES-GFP), which is normally confined to the cytoplasm. In the setting of nuclear membrane ruptures, NES-GFP appeared in the nucleus. They also observed cGAS at sites of nuclear membrane ruptures. In fibrosarcoma cells, they observed a 10-fold increase in nuclear membrane ruptures when the pore sizes in collagen matrices were reduced to 5–20 μm^2^. Ruptures appeared to occur in protrusions at the leading edge of the nucleus. Knocking down lamin A/C or lamin B2 in breast cancer cells with siRNAs increased the frequency of nuclear membrane ruptures.

Both Raab *et al*. [6] and Denais *et al*. [7] found that nuclear membrane ruptures in migrating cells were accompanied by an accumulation of fluorescently-labeled 53BP1, a protein that is recruited to double-stranded DNA breaks and to regions of low chromatin density [8]. In migrating osteosarcoma cells with nuclear membrane ruptures, Irianto *et al*. [8] observed increased numbers of γ-H2AX foci in the nucleus and DNA breaks based on electrophoretic ‘comet’ assays, as well as escape of DNA repair factors (Ku80, BRCA1) into the cytoplasm.

## Cytoskeletal–nuclear force transmission triggers nuclear membrane ruptures

The transmission of cytoskeletal forces to the nucleus increases the frequency of nuclear membrane ruptures. For example, Raab *et al*. [6] observed that increased force transmission to the cell nucleus as mouse dendritic cells migrate through dense extracellular matrices resulted in more nuclear membrane ruptures [6]. Also, an uncontrolled accumulation of contractile actin stress fibers over the cell nucleus [10] resulted in nuclear deformation and increased numbers of nuclear membrane ruptures. Conversely, Hatch *et al*. [11] showed that depolymerizing actin filaments with cytochalasin D or latrunculin – or inhibiting the actomyosin network with blebbistatin – reduced nuclear membrane ruptures in osteosarcoma cells with a knockdown of lamin B1. Additionally, Xia *et al*. [12] showed that blebbistatin suppressed nuclear membrane ruptures when A549 cells (with a lamin A knockdown) and osteosarcoma cells migrated through small pores [13]. In 2019, Cho *et al*. [14] also showed that blebbistatin suppressed nuclear envelope disruptions in the embryonic chick heart. Disrupting the ‘linker of nucleoskeleton and cytoskeleton’ (LINC) complex, either by reducing SUN protein expression or by disrupting nesprin–SUN protein interactions with a KASH domain, also reduced the frequency of nuclear membrane ruptures [11]. The LINC complex, located in the perinuclear space, is crucial for transmitting forces from the cytoskeleton to the nuclear lamina [15].

## Nuclear membrane ruptures in fibroblasts lacking all nuclear lamins

**Figure 1.**
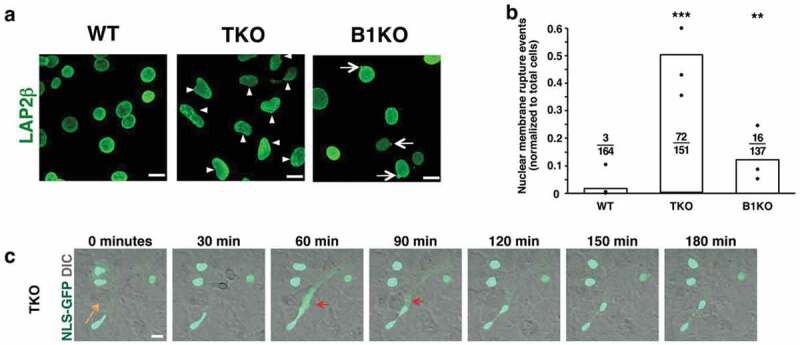
Abnormal nuclear shape and nuclear membrane ruptures in mouse embryonic fibroblasts (MEFs) lacking all nuclear lamins. (a) Confocal micrographs of *Lmna*^+/+^*Lmnb1^+^*^/+^*Lmnb2*^+/+^ (wild-type; WT), *Lmna*^–/–^*Lmnb1*^–/–^*Lmnb2*^–/–^ (triple knockout; TKO), and *Lmna*^+/+^*Lmnb1*^–/–^*Lmnb2*^+/+^ (*Lmnb1* knockout; B1KO) MEFs. Cells are stained with an antibody against LAP2β, an inner nuclear membrane protein (green). Arrowheads point to irregularly shaped nuclei; arrows point to nuclear blebs. Scale bars, 20 μm. (b) Bar graph showing numbers of nuclear membrane rupture events in WT, TKO, and B1KO MEFs. Black circles show the average number of nuclear membrane events (divided by numbers of cells examined) in three independent experiments. Numerical ratios show the total number of nuclear membrane rupture events in all three experiments divided by the total number of cells examined. **P < 0.001; ***P < 0.0001 by *χ*^2^ test. (c) Confocal micrographs showing a nuclear membrane rupture (red arrows) in a TKO MEF [expressing a nuclear-localized green fluorescent protein (NLS-GFP)] as it migrated across the field (orange arrow depicts the direction of migration). NLS-GFP is in green; the differential interference contrast (DIC) image is in gray. Scale bar, 20 μm. Reproduced with permission from Chen *et al*. [16].

Multiple studies have documented nuclear membrane ruptures when the nuclear lamina is weakened or compromised by reduced expression of nuclear lamins or by the expression of mutant nuclear lamin proteins [3–7,11]. In general, cells exhibiting nuclear membrane ruptures had nuclear protrusions or nuclear blebs. One possible interpretation of those studies is that the nuclear lamin fibrils in a structurally weakened or patchy fibrillar meshwork simply lacerate the nuclear membranes. If that were the case, one could imagine that a complete absence of a nuclear lamina might reduce the frequency of nuclear membrane ruptures (*i.e*., the nuclear membranes might be less prone to tearing by nuclear lamin fibrils). On the other hand, one could easily imagine that the nuclear membranes may be more prone to breaks caused by excessive stretching from cytoskeletal forces, resulting in frequent nuclear membrane ruptures. To investigate this issue, we [16] generated nuclear lamin–deficient (*Lmna*^–/–^*Lmnb1*^–/–^*Lmnb2*^–/–^) mouse embryonic fibroblasts (MEFs). The nuclei of these triple-knockout (TKO) MEFs were more irregularly shaped (Figure 1(a)) but nuclear blebs were absent. By transmission electron microscopy, the inner nuclear membranes in TKO MEFs cells were wavy, consistent with the absence of an underlying nuclear lamina. Nuclear pore complexes in the TKO MEFs were distributed asymmetrically [16], consistent with earlier observations by Guo *et al*. [17].

By live-cell microscopy, TKO MEFs expressing NLS-GFP exhibited frequent nuclear membrane ruptures (escape of NLS-GFP into the cytoplasm) [16]. Over a 50-h time frame, 23 of 151 cells had a nuclear membrane rupture, and the majority of those cells with ruptures had more than one cycle of nuclear membrane rupture and repair (Figure 1(b–c)) [16]. The nuclear membrane ruptures occurred spontaneously and in the absence of a nuclear protrusion or bleb. TKO MEFs rarely died during the 50-h period of live cell microscopy, but DNA damage (γ-H2AX foci) was significantly greater in TKO MEFs than in wild-type (WT) MEFs [16].

**Figure 2.**
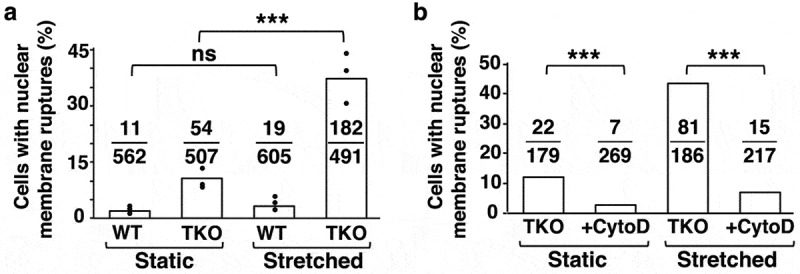
Effects of uniaxial stretching and actin depolymerization on nuclear membrane ruptures. (a) Bar graph showing percentages of wild-type (WT) mouse embryonic fibroblasts (MEFs) and triple-knockout (TKO) MEFs with nuclear membrane ruptures under static conditions and with uniaxial stretching. TKO MEFs lack all nuclear lamins. Black circles indicate percentages of cells with nuclear membrane ruptures in three independent experiments. Numerical ratios show the total number of cells with nuclear membrane ruptures in all three experiments over the total number of cells examined. ***P < 0.0005; ns, nonsignificant. (b) Bar graph showing that cytochalasin D reduces the percentage of nuclear membrane ruptures in TKO MEFs cultured under static conditions and with uniaxial stretching. Numerical ratios show the total number of cells with nuclear membrane ruptures over the total number of cells examined. ***P < 0.0001. Reproduced with permission from Chen *et al*. [16].

When TKO MEFs were subjected to mechanical stress (uniaxial stretching), the frequency of nuclear membrane ruptures increased (Figure 2(a)). The frequency of ruptures was reduced by cytochalasin D, both during static conditions and uniaxial stretching (Figure 2(b)). Cytochalasin D also reduced DNA damage [16]. Thus, despite an absence of a nuclear lamina and an absence of nuclear blebs, cytoplasmic forces remained important in triggering nuclear membrane ruptures. In parallel, we examined the frequency of nuclear membrane ruptures in *Lmnb1*-deficient MEFs (B1KO MEFs). B1KO MEFs have nuclear blebs (Figure 1(a)) but the frequency of nuclear membrane ruptures was lower than in TKO MEFs (Figure 1(b)). By live-cell fluorescence microscopy, we observed no evidence that the nuclear membrane ruptures in B1KO cells originated from a nuclear bleb, and the majority of ruptures occurred in cells without a nuclear bleb. Thus, nuclear blebs were not a useful predictor of nuclear membrane ruptures in either TKO or B1KO MEFs.

To gain insights into how different nuclear lamin proteins affect the frequency of nuclear membrane ruptures, we transfected TKO MEFs with expression vectors for prelamin A, lamin B1, and lamin B2 [16]. The expression of lamin B1 in TKO MEFs led to the emergence of nuclear blebs, but the frequency of nuclear membrane ruptures was reduced by about one-third [16]. Interestingly, however, the duration of ruptures was not reduced [16]. The expression of lamin B2 in TKO MEFs did not elicit nuclear blebs, nor did it change the frequency of nuclear membrane ruptures [16]. In TKO MEFs transfected with the prelamin A vector, mature lamin A was present at the nuclear rim, and around 40% of the lamin A-expressing cells had nuclear blebs. This result was not particularly surprising because we had previously observed a high frequency of nuclear blebs in keratinocytes that lacked lamin B1 and B2 but had robust expression of A-type lamins [18,19]. However, we were surprised by the impact of lamin A expression on nuclear membrane ruptures. Lamin A expression markedly increased the frequency of nuclear membrane ruptures in TKO MEFs, but the *duration* of ruptures was shorter (~30 min in the lamin A-expressing TKO MEFs *vs*. ~240 min in the nontransfected TKO MEFs) [16]. Why did lamin A increase the frequency of ruptures? The answer is not clear, but we suspect that the answer might relate to the distribution of different nuclear lamin proteins within the nuclear lamina. Recent studies revealed that lamin B1, which has a carboxyl-terminal farnesylcysteine methyl ester, is positioned immediately adjacent to the inner nuclear membrane [20], whereas the lamins A and C are normally positioned inside the lamin B1 meshwork (closer to the nucleoplasm). In lamin A–expressing TKO MEFs, the lamin A presumably lies adjacent to the inner nuclear membrane but, unlike lamin B1, is not tethered to the membrane by a farnesyl lipid anchor. Perhaps the untethered lamin A fibrils are more mobile and more prone to abrading the nuclear membranes, explaining the higher frequency of nuclear membrane ruptures. At the same time, the lamin A meshwork seems to facilitate membrane repair, given that the duration of nuclear membrane rupture was significantly shorter.

The very different effects of lamin B1 and lamin A expression on the phenotype of TKO MEFs demands a nuanced view of the relationship between nuclear membrane blebs and nuclear membrane ruptures. In TKO MEFs, both lamin B1 expression and lamin A expression resulted in the appearance of nuclear membrane blebs, but lamin B1 expression reduced ruptures while lamin A expression had the opposite effect. Also, lamin A expression reduced the duration of ruptures while lamin B1 did not.

## Are nuclear membrane ruptures physiologically relevant?

Our studies of TKO MEFs revealed frequent nuclear membrane ruptures and DNA damage [16], but the cells grew rapidly [18,19] and cell death was very rare, as judged by videomicroscopy [16]. The impressive vitality of TKO MEFs raised a provocative question: Are nuclear membrane ruptures innocuous in living animals or do they adversely affect cell vitality in the setting of physiologic or developmental processes in living animals?

To define the importance of nuclear membrane ruptures *in vivo*, we [16] proposed, in 2018, that investigating migrating neurons in the developing brain would be a reasonable place to start. Very recently, our suggestion was endorsed by another group [21]. For us, investigating the developing brain model was intriguing. First, neurons in the developing mouse brain (before E18.5) express *Lmnb1* and *Lmnb2* (the genes for lamin B1 and lamin B2) at high levels, but the expression of *Lmna* (the gene for lamin A and lamin C) is absent [22–25]. There is no reason to believe that the ‘natural deficiency’ of lamin A and lamin C in cortical neurons would impair neuronal migration, but we suspected that a deficiency of one additional nuclear lamin protein (either lamin B1 or lamin B2) might render neurons susceptible to nuclear membrane ruptures [26]. Second, the glial-directed migration of neurons from the ventricular zone (where neurons are born) to their final position within the cortical plate subjects neurons to mechanical stress. The migration of neurons is utterly dependent on nucleokinesis – a process by which cytoplasmic motors pull the nucleus forward (along microtubules) into the leading edge of the cell. After the nucleus is pulled forward, the trailing edge of the cell remodels, resulting in net forward movement of the neuron [27–31]. Multiple cycles of nucleokinesis, followed by remodeling of the trailing edge of the cell, results in saltatory movement of the neuron to its final position within the cortical plate.

Coffinier and coworkers discovered, using immunohistochemistry and BrdU birthdating experiments, that the glial-directed migration of neurons from the ventricular zone to the cortical plate is defective in both *Lmnb1*- and *Lmnb2*-deficient embryos [24,32]. The defective migration of neurons results in markedly abnormal layering of neurons in the cerebral cortex. Defective neuronal migration is likely caused by ineffective nucleokinesis. Because a deficiency of lamin B1 or lamin B2 reduces the integrity of the nuclear lamina, the cytoplasmic motors that normally pull the nucleus into the leading edge of the cell are ineffective. Rather than moving the nucleus, they simply ‘stretch out’ and deform the nucleus [24]. Misshapen nuclei are common in the cortical plate of *Lmnb1*^–/–^ and *Lmnb2*^–/–^ embryos [24]. In addition to defective layering of cortical plate neurons, there was another noteworthy finding in *Lmnb1*^–/–^ and *Lmnb2*^–/–^ embryos – fewer neurons and evidence of neuronal cell death [24]. Decreased cellularity of the cortical plate was striking in E18.5 *Lmnb1*^–/–^ embryos but subtle in E18.5 *Lmnb2*^–/–^ embryos [24,32]. *Lmnb1*^–/–^ and *Lmnb2*^–/–^ embryos die at birth, but forebrain-specific knockout models survive normally. Inactivation of *Lmnb1* or *Lmnb2* in the forebrain resulted in a progressive loss of neurons. By two months of age, forebrains in forebrain-specific knockout mice were very small and the numbers of viable neurons were markedly reduced. In two-month-old forebrain-specific *Lmnb1*/*Lmnb2* double-knockout mice, neurons were undetectable [24]. Thus, lamin B1 and lamin B2 are crucial for the postnatal survival of cortical neurons [24,32].

## Nuclear membrane ruptures, DNA damage, cell death, and tissue pathology in *Lmnb1*-deficient mice

In the setting of lamin B1 or lamin B2 deficiency, we hypothesized that the mechanical stress associated with nucleokinesis and cell migration could trigger nuclear membrane ruptures and lead inexorably to cell death. We investigated this hypothesis in cell culture models and in the developing cerebral cortex of mouse embryos. Our studies uncovered, for the first time, a link between nuclear membrane ruptures, neuronal survival, and tissue pathology [26].

**Figure 3.**
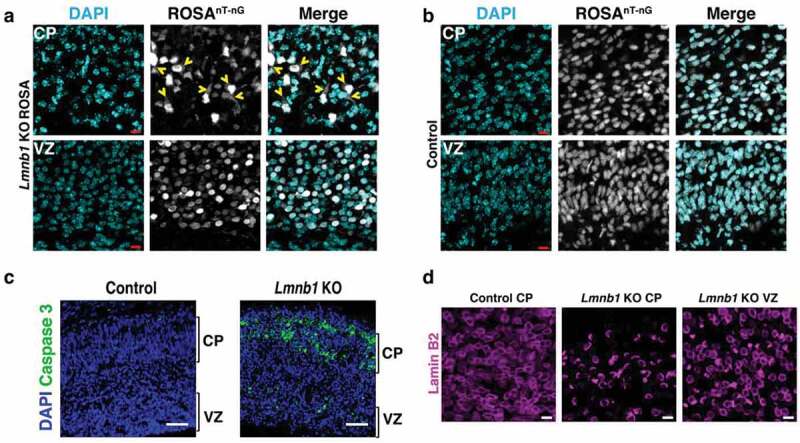
Nuclear membrane ruptures and cell death in the forebrain of E18.5 mouse embryos lacking lamin B1 expression in the forebrain. (a–b) Fluorescence micrographs of the cortical plate (CP) and ventricular zone (VZ) in the forebrain of (a) an E18.5 forebrain-specific *Lmnb1* knockout (KO) embryo harboring a ROSA^nT-nG^ transgene (*Lmnb1* KO ROSA) and (b) an E18.5 control embryo. The ROSA^nT-nG^ transgene encodes a fluorescent reporter that is normally confined to the nucleus. The fluorescent signal from the ROSA^nT-nG^ transgene is colored white. DNA was stained with DAPI (cyan). Yellow arrowheads point to *Lmnb1* KO ROSA neurons in which the fluorescent reporter had escaped into the cytoplasm (indicating a nuclear membrane rupture). Scale bars, 10 μm. (c) Confocal micrographs showing caspase 3 expression (green) in the forebrain of an E18.5 control embryo and a littermate forebrain-specific *Lmnb1* KO embryo. Caspase 3 is a marker of programmed cell death. DNA is stained with DAPI (blue). Scale bars, 50 μm. (d) Confocal micrographs showing the distribution of lamin B2 in forebrain neurons of a forebrain-specific *Lmnb1* KO embryo, revealing an asymmetric distribution of lamin B2 along the nuclear rim in CP neurons but not VZ neurons. Lamin B2 was distributed evenly in CP neurons of a control embryo. Scale bars, 10 μm. Images in all four panels are reproduced, with permission, from Chen *et al*. [26].

To explore the possibility of nuclear membrane ruptures in the developing brain, we bred *Lmnb1*-deficient mice harboring the ROSA^td-Tomato^ transgene [26], which encodes a nuclear-localized fluorescent protein. When we examined the cerebral cortex of E18.5 embryos, we found that the fluorescent marker had escaped into the cytoplasm in many cortical plate neurons, indicating nuclear membrane ruptures (Figure 3(a)). While there were numerous nuclear membrane ruptures in cortical plate neurons, no ruptures were observed in cells of the ventricular zone (where neurons reside before migrating to the cortical plate) (Figure 3(a)) [26]. Nuclear membrane ruptures were never observed in cortical plate or ventricular zone neurons of wild-type embryos (Figure 3(b)). We also performed immunohistochemical studies with an antibody against caspase 3, a marker of apoptotic cell death, to determine if nuclei of cells with ruptured nuclear membranes would be positive for caspase 3. In E18.5 *Lmnb1*-deficient embryos, caspase 3 staining was abundant in the cortical plate, whereas staining was negligible in the ventricular zone (Figure 3(c)) [26]. Caspase 3 staining was also negligible in neurons of wild-type embryos (Figure 3(c)).

In cortical plate neurons of E18.5 *Lmnb1*-deficient embryos, lamin B2 was mislocalized to one side of the nucleus, leaving a large segment of the nucleus completely devoid of nuclear lamins [26]. Remarkably, lamin B2 was distributed evenly in neurons of the ventricular zone (Figure 3(d)). We suspect that the asymmetric distribution of lamin B2 in *Lmnb1*-deficient neurons in the cortical plate was the result of mechanical forces imparted on the nucleus by nucleokinesis and neuronal migration [26]. We further suspect that the asymmetric distribution of lamin B2 in *Lmnb1*-deficient cortical plate neurons (and the complete absence of nuclear lamins along a substantial segment of the nuclear rim) is a key factor in their susceptibility to nuclear membrane ruptures and cell death.

## Nuclear membrane ruptures in cultured neurons derived from *Lmnb1*- and *Lmnb2*-deficient mice

**Figure 4.**
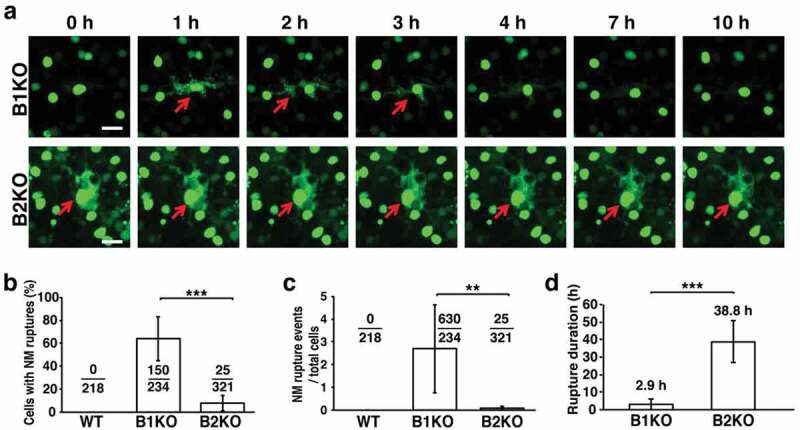
Nuclear membrane ruptures are frequent but are repaired quickly in cultured B1KO neurons, whereas ruptures are infrequent and prolonged (never undergoing repair) in B2KO neurons. (a) Live-cell fluorescence microscopy images of B1KO and B2KO neurons that express a nuclear-localized green fluorescent protein (NLS-GFP). Red arrows point to cells with nuclear membrane ruptures. Scale bar, 20 μm. (b) Percentages of neurons with nuclear membrane (NM) ruptures in five independent experiments. Numerical ratios show the total numbers of cells with NM ruptures over the total numbers of cells examined. ***P < 0.0001 as defined by a *χ*^2^ test. (c) NM rupture events in five independent experiments. Numerical ratios show the total numbers of NM ruptures over the total numbers of cells observed. **P < 0.001 as defined by a Student’s *t* test. (d) Average duration of NM ruptures. Repair of a NM rupture was *never* observed in a B2KO neuron; thus, the duration of 38.8 h represents the average length of time that ruptures were observed over the entire 50-h period of imaging. ***P < 0.0001 as defined by a Student’s *t* test. Reproduced with permission from Chen *et al*. [26].

We isolated *Lmnb1*-deficient (B1KO) neuronal progenitor cells, transduced them with NLS-GFP, cultured them as neurospheres, and allowed them to differentiate into neurons. The distribution of lamin B2 in cultured B1KO neurons was asymmetric, such that a large segment of the nuclear rim was devoid of lamin B2 [26]. By videomicroscopy, we observed multiple cycles of nuclear membrane rupture and repair in many B1KO neurons (escape of NLS-GFP into the cytoplasm followed by its return to the nucleus) (Figure 4(a)). Ruptures were observed in >60% of B1KO neurons (Figure 4(b)). During 50 h of observations, we observed 630 nuclear membrane ruptures in 150 B1KO neurons but no ruptures in wild-type neurons (Figure 4(c)). The mean duration of nuclear membrane ruptures in B1KO neurons was 2.9 h (Figure 4(d)), and one-third of the neurons died [26]. Consistent with findings in *Lmnb1*-deficient embryos, cultured B1KO neurons exhibited extensive DNA damage (numerous γ-H2AX foci in the cell nucleus) and stained positively for caspase 3 [26].

## Distinct nuclear membrane rupture phenotypes in lamin B1 – and lamin B2–deficient neurons

Finding nuclear membrane ruptures in cultured B1KO neurons prompted us to ask whether lamin B2 deficiency would elicit the same phenotypes. In considering this issue, it was difficult to make *a priori* predictions. On one hand, lamin B1 and lamin B2 are very similar proteins; they are ~60% identical at the amino acid level [33] and have virtually identical temporal and spatial patterns of expression in the developing brain [24]. On the other hand, our group has uncovered striking differences in the properties of the two proteins. First, lamin B1 is essential for a uniform distribution of lamin B2 along the nuclear rim, both in the cerebral cortex [24] and in the retina [34]. In contrast, the distribution of lamin B1 along the nuclear rim is not affected by a deficiency of lamin B2 [24,34]. Second, knock-in mouse embryos expressing *nonfarnesylated* lamin B1 manifest the same neurodevelopmental pathology observed in *Lmnb1*^–/–^ embryos, indicating that lamin B1’s farnesyl lipid anchor is required for neuronal migration and brain development. In contrast, knock-in mouse embryos that express a *nonfarnesylated* lamin B2 are healthy and free of pathology [35]. Third, in studies of ‘reciprocal *Lmnb1/Lmnb2* knock-in mice,’ we found that increased amounts of lamin B1 expression (driven by the *Lmnb2* promoter) could not prevent the neurodevelopmental abnormalities associated with lamin B2 deficiency [36]. Also, increased lamin B2 expression (driven by the *Lmnb1* promoter) did not reverse the neuropathology and early perinatal death associated with lamin B1 deficiency, although increased lamin B2 expression appeared to have a limited capacity to improve the survival of *Lmnb1*-deficient neurons [36].

To define the impact of lamin B2 deficiency on nuclear membrane integrity, we generated *Lmnb2*-deficient (B2KO) neurons expressing NLS-GFP [26]. Nuclear membrane ruptures were observed (Figure 4(a)), but the pattern of ruptures was distinct. First, the frequency of ruptures was lower in B2KO neurons than in B1KO cells (Figure 4(b–c)); only 7.8% of B2KO neurons had ruptures during 50 h of videomicroscopy (*vs*. >60% of B1KO neurons) [26]. Second, nuclear membrane ruptures in B2KO were *never* repaired, in contrast to the situation with B1KO neurons, where repeated cycles of nuclear membrane rupture and repair were frequently observed. The mean duration of ruptures in B2KO neurons was 38.9 h, 10-times longer than in B1KO neurons (Figure 4(d)). B2KO neurons with nuclear membrane ruptures invariably died, with fragmentation of the nucleus and progressive disintegration of the cell [26].

To test the capacity of lamin B2 to prevent nuclear membrane ruptures, B1KO and B2KO neurons were transfected with an inducible lamin B2 lentiviral vector [26]. Increased amounts of lamin B2 expression corrected the uneven distribution of lamin B2 along the nuclear envelope, such that the entire circumference of the nucleus in transfected cells was lined by lamin B2. By videomicroscopy, the frequency of nuclear membrane ruptures in lamin B2–transduced B1KO neurons was reduced by ~50% but was not abolished. Similarly, cell death was reduced but not abolished [26]. In contrast, overexpression of lamin B2 in B2KO cells eliminated nuclear membrane ruptures [26]. The fact that lamin B2 overexpression did not prevent ruptures in B1KO neurons underscores the findings that lamin B1 and lamin B2 have distinct functions in the nuclear envelope, as discussed earlier [36,37]. At this point, we do not fully understand why nuclear membrane rupture phenotypes are different in B1KO and B2KO neurons, nor do we understand why overexpression of lamin B2 fails to abolish nuclear membrane ruptures in B1KO cells. However, we suspect that lamin B1, which covers the entire nuclear rim, is more important than lamin B2 for maintaining the structural integrity of the nuclear envelope (explaining the high frequency of nuclear membrane ruptures in B1KO cells). Lamin B2 could be more important for nuclear membrane repair, given that we never observed repair of a nuclear membrane rupture in B2KO neurons. Perhaps lamin B2 serves as a ‘sealant’ to reinforce the lamin B1 meshwork, and that this ‘sealant function’ facilitates nuclear membrane repair. In any case, it ought to be possible, in future studies, to define the sequences in lamin B1 and lamin B2 proteins that underlie the distinct nuclear rupture phenotypes in B1KO and B2KO cells. For example, by comparing the capacity of lamin B1–lamin B2 chimeric proteins to reverse the nuclear membrane rupture phenotypes in B1KO and B2KO cells, one should be able to uncover the sequences responsible for the distinct nuclear membrane rupture phenotypes.

We suspect that the nuclear membrane rupture phenotype in B2KO neurons (infrequent ruptures, no rupture repair, and inexorable cell death) [26] provides an insight into the progressive loss of cortical neurons in forebrain-specific *Lmnb2* knockout mice [24,32]. Even if the frequency of nuclear membrane ruptures in lamin B2–deficient neurons is low, the inability to repair ruptures would be expected to lead to a progressive loss of viable neurons [26].

## External constrictive forces render B1KO neurons susceptible to nuclear membrane ruptures

**Figure 5.**
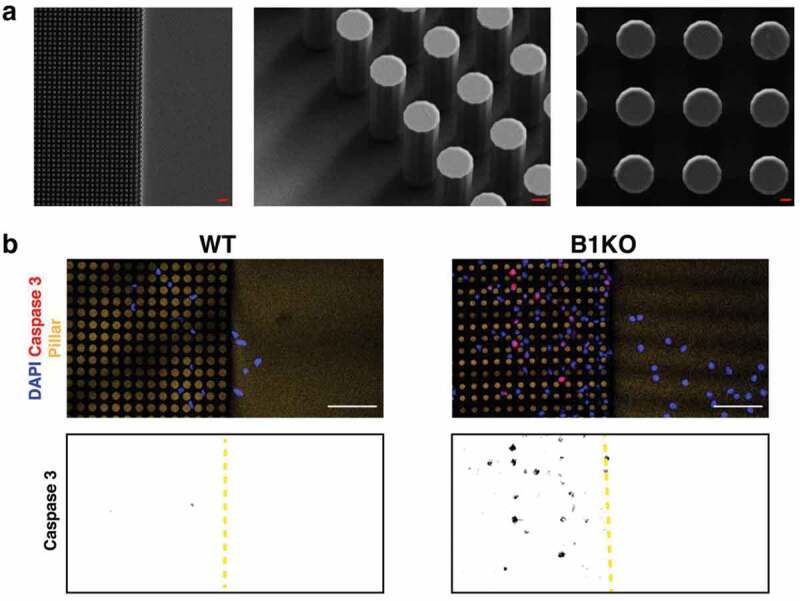
Migration of lamin B1 knockout (B1KO) neurons into a field of narrowly spaced pillars subjects cells to constrictive forces and results in widespread cell death. (a) (*left*) Scanning electron micrograph (SEM) of a silicon wafer (with one side flat and the other side containing uniformly spaced silicon pillars (8 μm in diameter; 22 μm in height; spaced 4 μm apart). Scale bar, 30 μm. (*middle*) Higher magnification SEM of the silicon pillars. Scale bar, 3 μm. (*right*) Higher magnification SEM of the silicon pillars. Scale bar, 2 μm. (b) Immunofluorescence microscopy of wild-type (WT) and B1KO neurons that had been stained with a caspase 3–specific antibody (a marker of apoptotic cell death; red). DNA was stained with DAPI (blue). Only one WT neuron exhibited caspase 3 staining, whereas 21 B1KO neurons had caspase 3 staining. Scale bars, 50 μm. The panels below show caspase 3 staining in black; the edge of the field of pillars is marked by a yellow dashed line. Reproduced with permission from Chen *et al*. [26].

We suspected that the nuclear membrane ruptures in cortical plate neurons of *Lmnb1*-deficient embryos were triggered by the forces of nucleokinesis and the constrictive forces on neurons as they traverse tight spaces between other cells in the cortical plate. To test the relevance of external constrictive forces on nuclear membrane ruptures, we plated NLS-GFP–expressing B1KO and wild-type neurons onto silicon wafers containing a field of silicon pillars (8 μm in diameter, spaced 4 μm apart) (Figure 5(a)) [26]. The migration of neurons into the field of pillars subjects cells to external constrictive forces. No ruptures were observed in the wild-type neurons when they entered the field of pillars, but we observed numerous nuclear membrane ruptures and widespread cell death when B1KO neurons entered the field of pillars (Figure 5(b)) [26]. Thus, external constrictive forces likely contribute to high frequency of nuclear membrane ruptures and cell death in the cortical plate of lamin B1–deficient embryos [24,26].

**Figure 6.**
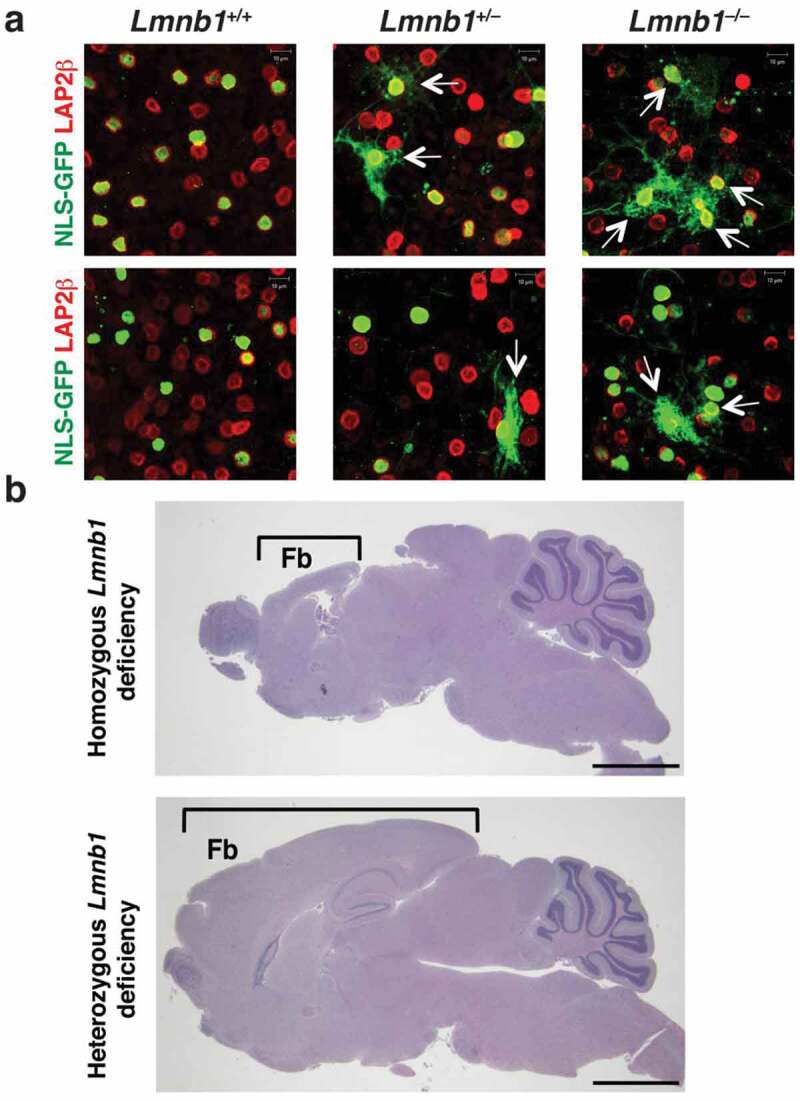
Identifying nuclear membrane ruptures in cultured cells does not always predict pathology in mouse models. (a) Confocal micrographs of NLS-GFP–expressing *Lmnb1*^+/+^, *Lmnb1*^+/–^, and *Lmnb1*^–/–^ neurons as they migrate away from cultured neurospheres, revealing nuclear membrane ruptures [escape of NLS-GFP (green) into the cytoplasm] in both *Lmnb1*^+/–^ and *Lmnb1*^–/–^ neurons. Ruptures were more frequent in *Lmnb1*^–/ –^ neurons than in *Lmnb1*^+/–^ neurons. The neurons were stained with antibodies against LAP2β (red), an inner nuclear membrane protein. White arrows point to cells with nuclear membrane ruptures. Scale bars, 10 μm. (b) Homozygous loss of *Lmnb1* in the forebrain in forebrain-specific *Lmnb1* knockout mice markedly reduces forebrain size; heterozygous loss of *Lmnb1* in the forebrain does not [24]. Brackets indicate the forebrain (Fb). Scale bars, 200 μm. Images in panel b reproduced, with permission, from Coffinier *et al*. [24].

Our findings in cultured B1KO neurons and neurons in *Lmnb1*-deficient embryos were concordant [26]. In both settings, nuclear membrane ruptures could be visualized *directly* (by visualizing the escape of a nuclear-localized fluorescent maker into the cytoplasm), and in both settings the ruptures resulted in DNA damage and cell death. Our experimental strategy – pursuing both cell culture studies and histopathologic studies in mouse embryos – was useful for defining the significance of morphological abnormalities in the cell nucleus (*e.g*., nuclear blebs) as well as functional defects (*e.g*., nuclear membrane ruptures). Inferring pathophysiologic significance to findings observed only in cultured cells is perilous. For example, a combined deficiency of lamin B1 and lamin B2 in cultured hepatocytes results in a very high frequency of nuclear blebs, but liver function tests and liver histology were normal in hepatocyte-specific *Lmnb1/Lmnb2* double-knockout mice [37]. Caution is also warranted in inferring pathophysiologic significance to nuclear membrane ruptures in cultured cell lines or cells harvested from mice and then studied *ex vivo*. For example, we had no trouble identifying nuclear membrane ruptures in *Lmnb1*^+/–^ neurons as the neurons migrated away from cultured neurospheres (Figure 6(a)), but we did not observe any neuropathology in mice that were heterozygous for *Lmnb1* deficiency (Figure 6(b)). It is possible that our cell culture conditions (plating neurons on polyornithine-coated plastic cell culture dishes) increased cytoskeletal forces on the nucleus [12] and contributed to nuclear membrane ruptures in cultured *Lmnb1*^+/–^ neurons. In any case, our observations provided an important lesson – that caution is warranted when one is tempted to infer pathophysiologic significance to findings observed only in cultured cells or only in *ex vivo* analyses of cells harvested from mice. To make sense of the cell culture findings, histopathologic studies in mouse models are crucial.

## Relevance of nuclear membrane ruptures outside of the central nervous system

**Figure 7.**
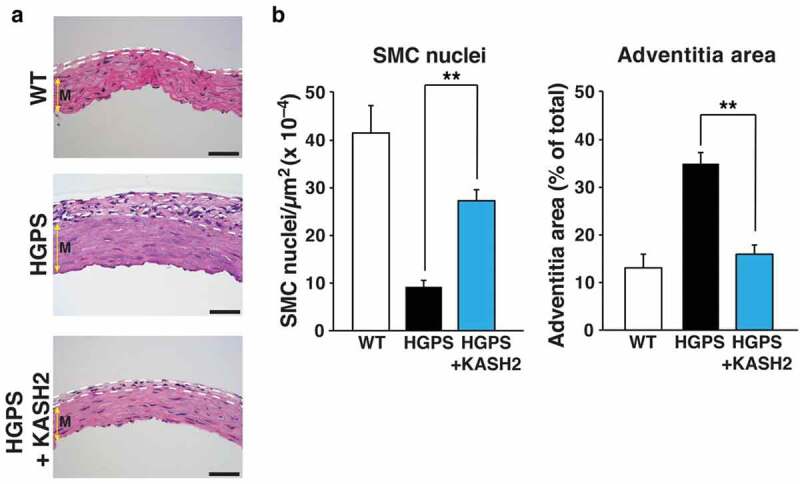
Expression of KASH2 in smooth muscle cells (SMCs) ameliorates aortic disease in a mouse model of HGPS. The HGPS mice (*Lmna*^G609G/G609G^ mice) were homozygous for the most common point mutation found in children with HGPS. KASH2 expression was from a *Cre*-activatable transgene (KASH2-EGFP); KASH2 expression was activated by an *Sm22-Cre* transgene. (a) Representative images of H&E-stained cross sections of the outer curvature of the ascending aorta in wild-type (WT) mice (*Lmna*^+/+^KASH2-EGFP^+^*Sm22-Cre*^+^), HGPS mice (*Lmna*^G609G/G609G^KASH2-EGFP^+^), and HGPS mice that expressed KASH2 in aortic SMCs (*Lmna*^G609G/G609G^KASH2-EGFP^+^*Sm22-Cre*^+^). Dotted white lines outline the adventitial layer of the aorta. Colored yellow arrow indicates the medial layer of the aorta (m). Scale bars, 50 μm. (b) Bar graphs depicting medial SMCs (nuclei per μm^2^) and adventitial area as a percentage of total area of the cross section. *n* = 6/group; **P < 0.001 as defined by a Student’s *t* test. Reproduced with permission from Kim *et al*. [47].

Nuclear membrane ruptures contribute to the neuronal cell death and neuropathology in lamin B1–deficient embryos [26], but we doubt that the relevance of ruptures is restricted to the developing brain. Sooner or later, we suspect that nuclear membrane ruptures will be shown to be important in the pathophysiology of multiple laminopathies, particularly those associated with cell death and overt tissue pathology. In recent years, there has been considerable interest in the arterial pathology of Hutchinson-Gilford progeria syndrome (HGPS) [38], a progeroid disorder caused by a mutant form of prelamin A (progerin) [39–41] that cannot be processed to mature lamin A [39–41]. Progerin accumulates at the nuclear rim and causes frequent nuclear blebs [39,42,43]. Histopathologic studies of the aorta in HGPS mouse models have revealed a striking loss of medial smooth muscle cells (SMCs) [44–46]. The loss of SMCs is associated with a compensatory increase in the thickness of the adventitial layer of the aorta; both the loss of medial SMCs and the adventitial thickening are progressive and was more severe in regions of the aorta subjected to the highest levels of stress [47]. In a 2018 paper, we [47] made a seminal observation about nuclear lamin gene expression in the aorta. In medial SMCs, we found that the expression of *Lmna* is quite high, whereas *Lmnb1* expression is very low. In HGPS mice, we observed very high levels of progerin protein in the medial SMCs of the aorta but very low levels of lamin B1 protein [47]. In contrast, both proteins were expressed at high levels in the endothelial cells of the arterial intima. We hypothesized that this combination – high levels of progerin but low levels of lamin B1 – rendered SMCs susceptible to injury in the setting of mechanical stress – and that it might be possible to prevent SMC death by blocking transmission of cytoskeleton forces to the nucleus. To explore that hypothesis, we examined the effects of disrupting the LINC complex in both progerin-expressing SMCs in cell culture and in the aortas of HGPS mice. To disrupt the LINC complex, we expressed the KASH (Klarischt/Anc-1, Syne Homology) domain of Nesprin2 (KASH2) [47] in SMCs, which interferes with Nesprin–Sun protein interactions in the perinuclear space and limits transmission of cytoskeletal forces to the nucleus. The hypothesis that interrupting force transmission to the nucleus would ameliorate disease phenotypes was confirmed – both in progerin-expressing SMCs in culture and in histopathologic studies of HGPS mice (Figure 7) [47]. In cultured progerin-expressing SMCs, KASH2 expression eliminated nuclear blebs and markedly reduced DNA damage. Moreover, when cells were subjected to uniaxial stretching, KASH2 expression improved cell survival [47]. To define the impact of KASH2 expression on aortic disease in HGPS mice, we [47]. bred HGPS mice harboring a transgene encoding a *Cre*-activatable KASH2 domain. Activation of KASH2 expression in aortic SMCs significantly reduced death of SMCs in the medial layer of the aorta and reduced the compensatory thickening of the adventitial layer (Figure 7) [47]. Given that KASH2 expression normalized nuclear shape in progerin-expressing SMCs in culture, prevented DNA damage, and improved survival in response to uniaxial stretching, it seems possible that nuclear membrane ruptures occur in SMCs and underlie the progressive loss of SMCs in the medial layer of the aorta in HGPS mice. This scenario is plausible, particularly since our electron microscopy studies [47] uncovered profound morphological defects in the nuclear membranes of medial SMCs in aortas of HGPS mice.

More recently, Earle *et al*. [21] investigated the relevance of nuclear membrane ruptures to myopathy in *Lmna*^–/–^ mice and gene-targeted mice harboring lamin A/C missense mutations known to cause myopathy in humans. In cultured myotubes (generated from myoblasts isolated from the mutant mice), they observed misshapen nuclei, DNA damage, and frequent nuclear membrane ruptures. In myofibers harvested from mutant mice, they found that expression of a KASH2 domain reduced DNA damage, consistent with our earlier studies [47]. They also found indirect evidence for nuclear membrane ruptures in myofibers harvested from mice (cGAS foci and the presence of heat shock protein 90 in the nucleus). Whether the expression of the KASH2 domain reduced nuclear membrane ruptures in diseased skeletal muscle beds or reduced tissue pathology was not examined. However, immunohistochemical studies of skeletal muscle biopsies from human patients with *LMNA*-related muscular dystrophies raised the possibility of DNA damage (as judged by a modest increase in the binding of a 53BP1-specific antibody binding to skeletal muscle biopsies). We suggest that future studies of mouse models of *LMNA*-related muscular dystrophies should take advantage of the ROSA^td-Tomato^ transgene [26] and examine, in a more direct fashion, the frequency of nuclear membrane ruptures in specific skeletal muscle beds under different experimental conditions. Directly assessing the frequency of nuclear membrane ruptures in skeletal muscle beds, combined with quantification of dystrophic changes in skeletal muscles, could add considerably to our understanding of the *LMNA*-related myopathies.
